# Effect of Genotype and Environment on Food-Related Traits of Organic Winter Naked Barleys

**DOI:** 10.3390/foods11172642

**Published:** 2022-08-31

**Authors:** Jordyn S. Bunting, Andrew S. Ross, Brigid M. Meints, Patrick M. Hayes, Karl Kunze, Mark E. Sorrells

**Affiliations:** 1 Department of Food Science and Technology, Oregon State University, Corvallis, OR 97331, USA; 2 Department of Crop and Soil Science, Oregon State University, Corvallis, OR 97331, USA; 3 Plant Breeding and Genetics Section, School of Integrative Plant Science, Cornell University, Ithaca, NY 14853, USA

**Keywords:** naked barley, organic, food, genotype, environment, genotype x environment, beta-glucan, water absorption, batter flow, protein, starch

## Abstract

This study aimed to understand how genetics and environment influence organic winter naked barley composition and functionality, and to identify traits that might effectively categorize basic physicochemical functionality of food barley. Across three years, two locations, and 15 genotypes, genotype significantly influenced all 10 food-related traits and was the dominant influence for three. Location significantly influenced eight traits and was dominant for three. Year significantly influenced all traits but was dominant only for one. Of the interactions location * year was the most influential and was the dominant effect for two traits. For all interaction terms where genotype was a component, the effect sizes were either small or non-significant suggesting that even with low leverage traits there is the potential for genetic gain by observing trait rankings across environments. Principal component analysis identified six traits that could serve to categorize basic physicochemical functionality of food barley. These were grain protein content, beta-glucan content, flour-water batter flow, water solvent retention capacity, time to peak viscosity of cooked flour, and hardness of cooked intact grains.

## 1. Introduction

Most barley produced in the world does not feed people [1], but there are many indications that it should be considered as a raw material for food production. This is because barley is rich in the soluble fiber mixed linkage β-glucan (BG) and is a source of other micronutrients associated with the health benefits of whole-grain cereals in general [2] and barley specifically [3]. Consequently, increased consumption of whole-grain naked-barley can help to reduce the incidence of diet-related diseases, such as cardiovascular disease, type-2 diabetes, and metabolic syndrome [4,5]. Accordingly, an understanding of the impacts of genetics (G) and environment (E) and their interactions (GxE) on organic barley composition is needed to facilitate increased use of organic barley as a food raw material, to assist breeders aiming to improve functional or nutritional value of organically grown barley, and for developing varieties specifically adapted to organic production. According to Massman et al. [6] organic crop management needs diverse rotations for purposes of better soil health and the breaking of pest, disease, and weed cycles. Barley is eminently suitable in rotations for regenerative, organic, and low-input systems as it requires less nitrogen than most crops and inhibits weeds via fast canopy establishment. Barley also matures early, and thus avoids heat and drought, especially in Mediterranean climates. Accordingly, in breeding grains for organic crop production grain quality needs to be assessed on grain grown under organic crop management.

Whole-grain naked, or hull-less, barley is preferred over hulled (covered) barley as a raw material for food because naked-barley does not require abrasive dehulling (pearling) to become palatable. When used in its entirety, naked-barley is a whole-grain according to accepted definitions [7,8,9]. Foods derived from whole-grain naked barley are more nutrient dense than those made with pearled or otherwise refined barley [10,11].

Physical hardness of barley kernels varies and impacts both milling performance and water absorption of the resultant flour. Milling harder barley kernels results in coarser flour particle size. Harder genotypes require longer pearling times [12] and are reported to have lower water uptakes as uncooked intact kernels [13]. Factors contributing to differential kernel hardness include BG and arabinoxylan contents, cell wall thickness, protein content, properties of the endosperm protein matrix, growing conditions [13,14,15,16], and allelic differences in the hordoindolines [16,17,18].

BG is the primary non-starch polysaccharide in barley grain and makes up 2.5 to 11% of the weight of non-shrunken barley kernels. Soluble BG can form viscous solutions and transient gels at low weight concentrations and these attributes influence functionality both in food processing and in the gut [3,19]. Barley protein content ranges from 7 to 25% (mean~12%) of kernel weight [20]. Protein content has been associated with grain hardness, starch structure, and kernel size. Accordingly, differences in protein content likely have downstream consequences on flour particle size, flour water absorption, and degree of starch damage [14,21,22].

Starch composition influences the characteristics of cooked barley foods [23]. There are known genotypes with normal (17 to 28% amylose), waxy (0 to 10% amylose) and high amylose (>30% amylose). Compared to barley with normal-starch, waxy barley has higher swelling power, higher pasting viscosity, and reaches peak viscosity more rapidly [14,23,24]. Waxy starch can be bred into barley genotypes by targeting alleles at the Waxy (*Wx*) locus using marker-assisted selection. These alleles simultaneously decrease amylose content and increase BG content [25]. Starch type and the presence or absence of amylases (e.g., from preharvest sprouting) both affect structural and sensory outcomes in food products [14,26].

Research on whole grain naked barley quality and physicochemical functionality aims to improve nutritional and sensory attributes of whole grain barley foods. However, unknowns exist. For example, what are the most relevant physicochemical traits that can be used to characterize basic food functionality [14,27]. Absence of an objective classification schema beyond hulled/hull-less, colored/non-colored, and waxy/non-waxy leads to different barley lots in the food market being diverse in composition and physicochemical functionality [14,28] leading to problems in processing. Objective phenotypic validation of waxy vs. non-waxy status is also needed. Food barley breeding programs also need a set of agreed upon traits to categorize physicochemical functionality [14]. Accordingly, understanding the roles of G and E on organic naked food barley quality would be useful for breeders and organic farmers. Environment can also detrimentally alter starch or flour pasting properties when pre-harvest sprouting occurs [26,29].

We addressed these research questions: (1) How do G and E combine to determine the magnitudes of a chosen set of organic barley food quality traits and are the G and E effects different when observed in organic vs. non-organic systems? (2) What is a minimum number of traits that could usefully characterize the overall physicochemical functionality of naked food barley?

## 2. Materials and Methods

### 2.1. Materials

Fifteen naked barley genotypes (advanced breeding lines and released varieties) were grown at two rainfed locations over three harvest years (2018, 2019, 2020: Table S1). Locations were Corvallis OR and Freeville NY. Corvallis has a Mediterranean climate of wet winters and dry summers and is on average warmer (especially in winter) and slightly wetter than Freeville. Freeville has a more humid summer climate and rainfall more evenly spread throughout the year (Tables S2 and S3 and Figures S1 and S2). Plants headed earlier in Corvallis but harvest dates were similar and grain yield per hectare was comparable between the locations (Table S1). Of the 15 genotypes 11 had obligatory winter growth habits and four were facultative (Table 1). Twelve genotypes were reported to be non-waxy and three were reported to be waxy. Samples had mostly white seed coat color (Table 1).

Trials were grown on certified organic land in a randomized complete block design. All trials were planted in October of the year prior to harvest (Table S1). Corvallis research plots were planted, maintained, and harvested by the Oregon State University Barley Breeding Program. Plots were 9.3 m^2^ with six rows at 0.18 m spacing and seeded at 100 g/plot. Stutzman’s 8-2-4 organic chicken blood/feather meal (Stutzman Environmental Products, Inc. and J & D Fertilizer, Ltd., Canby, OR, USA) was applied at rate of 56 kg.ha^−1^ in the spring. Freeville trial plots were planted, maintained, and harvested by Cornell University Small Grains Project. Plots were 3.2 m^2^ with six rows at 0.18 m spacing and seeded at 68 g/plot. Plots did not receive supplemental fertilization. After harvest, grain was stored in plastic tubs (Corvallis) or paper bags (Freeville). After being received, the grain was stored at −23 °C for 14 days and allowed to thaw for at least three days at room temperature. Grain was cleaned of excess hulls in a head thresher (Precision Machine Co., Inc., Lincoln, NE, USA) until samples were at least 90% hull free. Cleaned grain was stored at room temperature in closed zip-closure plastic bags until needed. Reagents were analytical grade or better and purchased from Sigma-Aldrich (St. Louis, MO, USA). β-glucan assay kits were purchased from Megazyme International Ireland (Bray, Ireland).

#### Removal of Samples with Suspected Pre-Harvest Sprouting

Rapid Visco Analyzer (RVA) was used to identify samples impacted by pre-harvest sprout (PHS). Samples with peak RVA viscosities below 3000 cP were classified as sprouted because starch functionality was considered impaired below this value. For example, Zhou et al. [30] reported 11 of 12 lines of ungerminated barley to have RVA peak viscosities of >3000 cP when using a conversion factor of 10 from Rapid Visco units to cP [31], or all over 3000 cP if using a conversion factor of 12 [32]. The experimental design called for 12 entries for each analyzed trait from all 15 genotypes (2 biological replicates * 2 Locations * 3 years: *n* = 180). There were up to four missing entries per trait for the full data set (Table S2). There were up to 22 missing entries per trait when PHS affected samples were removed (Table 2).

### 2.2. Methods

Hardness index (HI) and moisture content were determined using a Perten 4100 Single Kernel Characterization System (SKCS) (Perten Instruments, Inc., Springfield, IL, USA). The method was adapted for barley grain from AACC-International (AACCI) Approved Method 55-31.01 [33]. One hundred fifty seeds from each sample were prepared by removing broken and shriveled seeds, and seeds with remaining hulls. The 150 seeds were individually crushed and the collective HI calculated by a SKCS machine algorithm [34,35].

Grain protein (12% moisture basis [mb]) was measured using near infrared reflectance spectroscopy (NIRS) (Infratec 1241 Grain Analyzer, FOSS, Laurel, MD, USA) using AACCI Approved Method 39-25.01 [36]. Grain was milled into whole-grain flour using a Perten Laboratory Mill 3100 (Perten Instruments, Hägersten, Sweden) with a 0.8 mm screen. Whole-grain flour was stored at room temperature and away from light in closed zip-closure plastic bags.

BG% (dry weight basis [db]) of whole grain barley flour was determined using the Megazyme assay procedure (Megazyme International Ireland, Bray, Ireland) using AACCI Approved Method 32-23.01 [37]. Water solvent retention capacity (W-SRC [%]) was measured on whole-grain barley flour using deionized water using AACCI Approved Method 56-11.02 [38]. Of the four SRC solvents only water was used. Prior work in our facility had shown problems with pellet sedimentation with the other SRC solvents when testing whole-grain naked barley flour [39]. Drakos et al. [40] reported no such issues in a study using a commercial barley flour. We speculate that that flour was probably milled from pearled barley and not whole-grain naked barley, which may account for the discrepancy.

Batter flow distance was measured on a 33.3% *w*/*w* batter of whole-grain barley flour in water (100 g flour [as-is] in 200 g water, i.e., 200% hydration based on bakers’ percentages) using a Bostwick Consistometer (CSC Scientific Company, Inc., Fairfax, VA, USA). A timer set to 20 min was started immediately when the flour and water came in contact. Twenty minutes was chosen based on the standard hydration time of the SRC test [38]. Flour and water were hand mixed vigorously with a fork for 1.0 min. The required outcome was a batter with smooth consistency with no lumps of dry flour. Fifteen sec vigorous hand mixing with a fork was also done at 5, 10, and 15 min elapsed time, and immediately prior to transfer of the batter to the Bostwick reservoir. The Bostwick trapdoor was triggered, and the flow distance (cm) was measured at 30 s. Results were recorded according to a modified version of the American Society for Testing and Materials protocol ASTM F1080–93, 2013 [41] where the result is averaged to the nearest 0.25 cm by taking the maximum flow distance (usually in the center of the trough) and adding this to the minimum distance (usually at one edge of the trough) and dividing the result by two.

RVA peak viscosity, breakdown, and time to peak viscosity of flour-deionized water suspensions were measured with an RVA-4500 (Perten Instruments, Hägersten, Sweden) using a modification of AACC International Method 76-21.01 [42]. The shortened time/temperature profile of Crosbie et al. [43] and 4.0 g of whole-grain flour (14% moisture basis) was used, i.e., weight of the tested flour sample with a moisture content different from 14% was equivalent in terms of the dry weight content of 4.0 g of flour with a moisture content of 14%. Starch type (waxy, non-waxy) information was supplied by the breeders and further verified through RVA testing. 

Whole grain cooked yield was measured using ~105 g of dry uncooked grain (as-is basis) weighed accurately. Grain was placed in a two liter All Clad saucepan (All Clad, Millville, NJ, USA) and then placed onto an induction burner (IWA-2500, Iwatani, Houston, TX, USA) set at Cooking Power Level 3 (out of 9) for a fixed time of 60 min. This power setting maintained the cooking water at a gentle rolling boil for the duration. Grain yield was measured by straining the cooked grain through a mesh strainer, shaking it for 10 s in the strainer, then allowing it to sit in the strainer for 50 more seconds. Cooked grain was then weighed. Cooked grain yield was calculated as the final cooked mass/initial uncooked mass X 100. 

Cooked whole grain hardness was measured using a TA.XT Plus texture meter with a back extrusion attachment (Texture Technologies Corp., Hamilton, MA, USA). The back-extrusion method was modified from Reyes and Jindal [44]. Force was reported in grams. The back-extrusion cell was a plexiglass cylinder, 10 mm thick, 100 mm deep, with 50 mm inside diameter. An aluminum flat disc probe, 45 mm diameter and 5 mm thick, centered in the cell, was used for compression. Cooked grain hardness was measured immediately after recording cooked-grain yield. The back-extrusion cell was filled, but not packed, with cooked grains, which were leveled with the top of the cell. Back-extrusion was performed as follows: compression speed, 2 mm/s; probe distance 75 mm; data recording commenced once the probe sensed a force of 25 g. The average steady state extrusion force was calculated by averaging the force data once steady state extrusion had commenced. In practice this was the average force between 75 and 90 s of elapsed time.

### 2.3. Statistical Analyses

All analyses were performed using two biological replications per location and one technical replication, unless otherwise noted. Multifactor and one-way analyses of variance and correlation analyses were carried out. Post hoc means comparisons were calculated using Tukey’s HSD or Student’s *t*. Mean-centered and scaled principal component analysis (PCA) was used to study interrelationships between physical characteristics, composition, and functionality tests. Analyses of variance and correlations were performed using R (R Core Team, Vienna, Austria). PCA used JMP v15.1.0 (SAS Institute Inc., Cary, NC, USA). Significance was set at a probability level of *p* ≤ 0.01.

## 3. Results and Discussion

### 3.1. Summary Statistics and Missing Entries

The chosen genotypes were variable in grain composition and pasting properties (Table 2). For example, grain protein ranged from around 6 to over 18% and BG content from around 3 to over 8%. Removing PHS affected samples had the greatest impact on RVA viscosities and cooked grain hardness: the traits with gelatinized starch. Changes in range, mean, and standard deviation for these traits were primarily a result of increased minimum values (Table 2 and Table S3). PHS was concentrated in five genotypes: DH140490 (waxy), 10.0662 (waxy), 10.0655 (waxy), DH140394, and VA15H-79WS. This result gives rise to the speculation that these five genotypes, and waxy naked-barley genotypes more generally, may be more susceptible to PHS. This requires further investigation.

### 3.2. Influence of Genotype, Location, and Year and Their Interactions on Food Traits

Three-way ANOVA was used to study the effects of genotype, location, and year on the chosen traits (Table 3). Overall model *r*^2^ values indicated good model fits for all traits. All three main effects had statistically significant influences on all traits except that location was non-significant for BG content and for cooked grain yield. Genotype had a significant influence on all 10 traits but was the dominant influence (as judged by the largest *F*-value within a trait) for just three: BG content, RVA peak time, and cooked grain yield. Location was the dominant effect for HI, W-SRC, and batter flow and had a substantial effect on RVA breakdown. Year was the dominant effect only for RVA breakdown, but year also had substantial effects on protein and on both cooked grain yield and hardness.

Most interactions had statistically significant influences on the measured traits (Table 3). In many cases, even when they were statistically significant, the interaction effect sizes (*F*-values) were much smaller than those of the main effects. There were notable exceptions. For example, location * year was the dominant effect for protein content and cooked hardness and had a substantial influence on HI, batter flow, and RVA breakdown and peak time. One could speculate that the large location * year interactions could result from the substantially drier conditions in Corvallis as well as the marked rain deficit February through May in Corvallis in 2020 (Figure S1). Of the interactions with genotype as a component, genotype * location, genotype * year, and genotype * location * year, none were the dominant effect for any trait. This suggests that genotypes ranked similarly across environments.

#### 3.2.1. Hardness Index

The waxy genotype DH140490 was the hardest and the non-waxy genotype Buck the softest (Table 3). Mean genotype HI values placed all barley grain in a hardness milieu that describes intermediate to moderately hard wheats. However, the overall range indicated that some individual samples were within the range of soft wheats with HI < 40 (Table 2). This aligns with the findings of Fox et al. [21], under non-organic crop management who indicated that most the grain of most of the barley cultivars in their study would be considered equivalent in hardness to medium to soft wheats with some individual samples being hard. Throughout we have presumed that if organic management is not specified in the cited papers that crop management was “conventional” or non-organic. Freeville samples were on average softer than Corvallis samples from the 2018 and 2020 harvests (Freeville 34.5 and 48.8, respectively and Corvallis 66.1 and 63.7 resp), but harder from the 2019 harvest (Freeville 53.9 and Corvallis 47.1). This accounted for the large and significant location * year interaction (Table 3). One-way ANOVA showed that the waxy genotypes on average were significantly harder (*p* = 0.001: Mean HI = 61.0) than non-waxy genotypes (Mean HI = 51.8). HI was found to be weakly but significantly correlated with BG content (*r* = 0.29, *p* = 0.0002), a result supported by literature based on both non-organic [13,14,45,46,47] and organic [39] management. HI of non-waxy genotypes had a weak relationship with BG content that approached significance (*r* = 0.19, *p* = 0.028). For waxy genotypes there was no significant relationship between HI and BG content.

#### 3.2.2. Protein Content

Mean protein contents of the genotypes varied between 8.8 and 11.4% (Table 3). The two locations, although significantly different from each other statistically, were only 0.5% different in mean protein content. The biggest influence on the overall range of protein contents appeared to be the low protein of the 2018 samples. Consistent with the dominant effect of the location * year interaction, Freeville had on average higher protein than Corvallis in 2018 and 2019 (Freeville 10.2 and 11.2%, respectively and Corvallis 7.6 and 10.7%, respectively) but had lower protein in 2020 (Freeville 8.4%, Corvallis 13.6%). Choi et al. [48] and Zhang et al. [49] found environmental effects dominated for protein content in trials with 17 genotypes, 8 locations, and 2 years, and 10 genotypes, 8 locations, and 2 years, respectively. Tamm et al. [50] ascribed changes in protein content in four naked and two covered spring-planted barleys to location in one trial and genotype * year in a second. All three cited trials were grown under non-organic conditions.

#### 3.2.3. Mixed Linkage Beta-Glucan

Consistent with the dominant effect of genotype on BG content (Table 3), waxy genotypes had the highest mean BG contents that ranged narrowly between 6.8 and 6.9%. Non-waxy genotypes varied in BG between 4.0 and 5.5%. One-way ANOVA confirmed that mean BG contents of waxy genotypes were significantly higher than those of the non-waxy genotypes (*p* < 0.0001). Within the non-waxy genotypes #STRKR, Buck, DH133535, DH140394, and VA15H-79WS were in the lowest BG group and 1_4, 10.1154, 10.1986, DH133529, and DH133783 were in the highest BG group. 2020 had the lowest mean BG content but was only 0.6% lower than 2019, the year with the highest BG content. A separate ANOVA was performed using only the non-waxy genotypes to determine whether it was solely waxy genotypes that were responsible for the large and significant genotype effect. Genotype remained the dominant influence on BG content in the absence of the waxy genotypes (*F*-value = 38.0, *p* < 0.0001), again followed by year as the second most influential factor (*F*-value = 17.2, *p* < 0.0001). These results align with the studies of Choi et al. [48] and Zhang et al. [49] who indicated that genotype was the dominant factor affecting BG content under non-organic conditions. Furthermore, Choi et al. [48] showed significant genotype * year, genotype * location, location * year, and genotype * location * year interactions as did this study. However, in our study the small effect sizes of the interaction terms with respect to the main genotype effect indicated that the interactions were subsidiary.

#### 3.2.4. Water-SRC

Waxy genotypes were statistically grouped and had significantly higher mean W-SRC values than all but one of the non-waxy genotypes (DH133783: Table 3). Waxy genotype values ranged from 122.4 to 126.6%. Non-waxy genotypes, except for DH133783, ranged between 99.7–106.9% (Table 3). Meints et al. [39] also found waxy genotypes to have high W-SRC values when grown under organic conditions. Non-waxy genotype DH133783’s high water absorption may have been related to its combination of high HI and moderately high protein and BG contents (Table 3). Samples from Corvallis had on average 9.6% higher W-SRC than Freeville. This effect on W-SRC may have been related to Corvallis samples having on average both higher HI and higher protein contents. HI and protein content were moderately and weakly, respectively, and positively correlated with W-SRC (HI: *r* = 0.50, *p* < 0.0001; Protein: *r* = 0.24, *p* < 0.0001). The moderate positive correlation between HI and flour water absorption may have been anticipated. It is aligned with common knowledge of this relationship in wheat. It is also inferred from the studies of Lee et al. [51] that showed that finer grinding increased barley flour starch damage and water absorption, and of Nair et al. [15] who showed increased protein adhesion to starch granules in harder barley kernels. Overall, BG content was moderately and positively correlated with W-SRC (*r* = 0.61, *p* < 0.0001) reflecting the well-established ability of water-compatible non-starchy polysaccharides to absorb water.

#### 3.2.5. Batter Flow

Waxy genotypes had the shortest batter flows, except for the non-waxy genotype DH133783, which also had a short batter flow and was statistically grouped with the waxy genotypes (Table 3). The arguments above for the higher W-SRC of the non-waxy DH133783 are also valid for its shorter batter flow when compared to the other non-waxy genotypes. The waxy-plus-DH133783 vs. non-waxy separation accounted for the moderately large effect size for genotype. Of the other non-waxy genotypes Buck had the longest batter flow consistent with its overall lower HI, and protein and BG contents (Table 3). Location was the dominant effect and Freeville samples had longer mean batter flows than samples from Corvallis (Table 3). Although mean Freeville batter flows (12.2, 8.3, and 12.2 cm in 2018, 2019, 2020, respectively) were longer in all years than Corvallis (8.5, 7.7, and 7.4 cm in 2018, 2019, 2020, respectively) the relative closeness of the 2019 values seems to account for the moderately large effect size for the location * year interaction. Batter flow had a significant, negative, and strong linear relationship with W-SRC (*r* = −0.81, *p* < 0.0001, [*r*^2^ = 0.66]). However, the relationship was clearly curvilinear, both by observation and by examination of the residuals around the linear line of best fit (data not shown). A power law curve gave the best fit (*r*^2^ = 0.80). Batter flow also had significant negative correlations with HI, protein content, and BG content (*r* = −0.59, −0.43, and −0.68, respectively *p* < 0.0001). These associations are consistent with the concept that increasing concentrations of protein and BG, and arguably higher starch damage from milling harder kernels, would increase water absorption and hence decrease batter mobility (flow).

#### 3.2.6. RVA Paste Viscosities

For RVA peak and breakdown viscosities, the effect sizes were similar for genotype, location, year, and the location * year interaction (Table 3). One-way ANOVA showed that peak viscosities did not differ significantly between waxy and non-waxy genotypes (*p* > 0.01). Contrary to the literature, the waxy genotypes tested in this study did not have the highest peak viscosities [14,23,24]. Indeed, the non-waxy genotype DH133529 had the highest peak viscosity and the waxy genotype DH140490 had the lowest. We doubt that the discrepancy between our results and those gathered largely under non-organic conditions are a function of the different crop management systems but rather, are a function of the spectrum of genotypes observed in this study. A study using the same genotypes grown under organic and non-organic conditions adjacent to each other would clarify this question.

Breakdown of viscosity under shear was significantly larger for waxy genotypes (*p* < 0.0001). This is arguably because of the quicker disintegration of the more fragile, highly swollen waxy granules during cook-up and is related to the significantly larger *absolute* breakdown values of waxy genotypes 10.0655 and 10.0662 (Table 3). The waxy genotype DH140490 seemed to break this association with a middle ranked absolute breakdown viscosity value (Table 3). However, re-calculation of the breakdown as a *proportion* of peak viscosity showed that DH140490’s proportional breakdown was 51.2% of peak viscosity and that all three waxy genotypes had proportional breakdown exceeding 50% of peak viscosity. No non-waxy genotype had breakdown exceeding 42.5% of peak viscosity. One-way ANOVA showed the mean proportional breakdown of the waxy genotypes (53.4%) to be significantly (*p* < 0.001) greater than mean non-waxy proportional breakdown (39.8%).

#### 3.2.7. RVA Peak Time

Peak time had a very large genotype effect followed by a large but subsidiary effect of location*year (Table 3). Waxy genotypes had shorter mean peak times (3.3 min) which were statistically separated from the non-waxy genotype peak times (5.0 min: *p* < 0.0001). Summary statistics of measured traits including samples with pre-harvest sprouting (Table S3).

To determine whether it was solely the waxy genotypes responsible for the large genotype effect, a separate ANOVA was performed on the non-waxy genotypes. Genotype still had a significant effect (*F*-value = 8.4, *p* < 0.0001) on peak time, but the effect size was decreased by two orders of magnitude. Location * year (*F*-value = 91.1, *p* < 0.0001) had the largest effect on the peak times of non-waxy genotypes, suggesting insufficient genetic variability to influence the RVA peak time via breeding and selection within the non-waxy genotypes tested in this study. These observations confirm that RVA peak time is strongly determined by waxy/non-waxy status [23]. The short RVA peak times of waxy types are potentially diagnostic of waxy status when starch type is otherwise unknown. DH140490 had the shortest peak time (Table 3). We contend that this, in conjunction with its high proportional paste viscosity breakdown (see Section 3.2.6.), confirms its waxy status. Additionally, although the non-waxy genotype DH133783 grouped with the waxy types for W-SRC and batter flow (Table 3) its long peak time and low proportional RVA breakdown (41%) indicated that it was indeed non-waxy. A combination of RVA peak time of <4.0 min and proportional breakdown of >50% when using the shortened RVA profile [43] has promise as a robust indicator of waxy vs. non-waxy status of barley flour in the absence of other genetic or analytic data.

#### 3.2.8. Cooked Grain Yields and Hardness

Overall, there was a relatively small range of mean cooked grain yields (252.5 to 279.4%: Table 3) and provided little information on which factors systematically affected cooked grain yields. BG content and RVA peak viscosity both had weak but significant (*p* < 0.01) positive correlations with cooked grain yield (*r* = 0.31 and 0.32, respectively). Waxy genotypes had mean cooked grain yields indistinguishable from non-waxy types (264 and 269%, respectively, *p* = 0.014). Year significantly affected cooked grain yield with an *F*-value in the same order of magnitude as genotype (Table 3). 2020 had the highest mean cooked grain yield but was only marginally higher than 2018 and 2019 (Table 3).

The non-waxy line DH133783 had the hardest cooked texture, likely due to high protein content combined with non-waxy starch (Table 3). Waxy genotypes had lower cooked whole grain hardness, except for 10.0662 that had moderately hard cooked texture. The observation that two of the three waxy genotypes had softer cooked hardness is aligned with the findings of Klamczynski et al. [52] who showed that cooked hardness of non-organic lightly dehulled covered waxy barley grain was softer than lightly dehulled covered non-waxy barley grain. It is not clear from the data why the cooked texture of 10.0662 was as hard as it was. Correlation analysis showed that cooked hardness was moderately and positively correlated with protein content (*r* = 0.52, *p* < 0.0001), weakly and negatively correlated with BG content (*r* = −0.21, *p* = 0.0075), and weakly and negatively correlated with both absolute and proportional RVA breakdown (both: *r* = −0.35, *p* < 0.0001). Klamczynski et al. [52] also found that protein content was positively correlated with cooked grain hardness when testing their lightly dehulled covered barleys. Our findings are moderately consistent with the findings that flour pastes with larger RVA breakdowns have been associated with softer cooked wheat-flour noodles [53] and softer cooked rice [54]. Corvallis samples had overall harder cooked textures than Freeville and much harder cooked textures in 2020. However, samples from Freeville were harder in 2018 and 2019 accounting for the dominant location * year effect.

### 3.3. Principal Component Analysis

**Figure 1:**
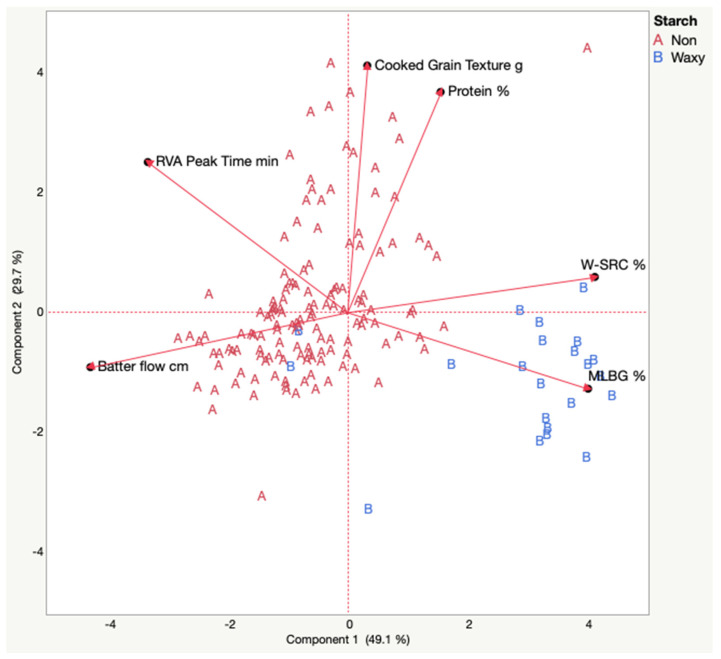
Mean-centered and scaled principal component biplot of 6 selected naked barley traits. A = non-waxy genotypes. B = waxy genotypes. MLBG = mixed linkage beta glucan. W-SRC = water solvent retention capacity.

In PCA of all ten measured traits, PC1 and PC2 accounted for 58.6% of the variance in the data. Traits were removed stepwise by sequentially removing the trait with the lowest cosine^2^ value and hence the lowest leverage in explaining the variance in the plane of PC1 and PC2. The traits removed were, in order, RVA peak viscosity, cooked grain yield, HI, and RVA breakdown. This led to PCA plots where PC1 and PC2 accounted for, respectively 63.4, 70.4, 75.3, and 78.9% of the variance, with 9, 8, 7, and 6 traits retained. The removal of HI was interesting as Baik and Ullrich [14] considered kernel texture to be very important to processing. What we retained though, were parameters that were the functional *outcomes* of milling kernels with varied textures (W-SRC, batter flow). Further reduction to 5 traits differed when either batter flow or W-SRC (these traits covaried), or protein (lowest cosine^2^ at six traits) were removed. In these cases, PC1 and PC2 accounted for 78.4, 80.6, and 83.9% of the variance, respectively. From a pragmatic perspective we selected six traits that are known to be useful to breeders and to the trade (Figure 1). These traits covered key elements of grain composition, (protein and BG), two pre-cooking functionality traits related to kernel texture (batter flow and W-SRC), and two cooked traits (RVA peak time and cooked hardness). In the six-trait PCA, PC1 was primarily aligned with vectors for high W-SRC, MLBG, and short batter flow going from left to right. PC2 was aligned with increased cooked hardness and higher protein content going from bottom to top. RVA peak time had a vector at nearly 45 degrees to both PCs. Waxy genotypes were mostly clustered to the right and in the lower right quadrant, as result of their generally higher BG content, W-SRC, shorter RVA peak times, and shorter batter flows. There was no obvious clustering of locations or years in PC1, PC2, or PC3 (PC3 data not shown). These results indicate that these six traits could be sufficient to characterize an unknown barley sample with respect to basic physicochemical functionality.

## 4. Conclusions

We conclude that genotype, location, crop year, and the interactions of crop year and location are significant influencers of organic naked barley grain composition and functionality. The results show that the general trends were like those reported for barley grain grown under non-organic management. This suggests that for grain traits, assessing them on non-organically managed crops would be sufficient to assess changes to the traits related to G or to E factors other than management. However, when breeding for organic or other low-input production systems important field traits such as fast canopy infill and resistance to seed and seedling disease pressures need to be assessed appropriately and the grain quality could be assessed under the correct management.

Genotype had a statistically significant influence on all ten food traits (Table 3). However, it was not always the dominant influence. For example, location dominated kernel HI, W-SRC, and batter flow suggesting reduced or nonexistent leverage to affect these traits via breeding. Nonetheless, that observation that all the interaction terms that included “genotype” were either non-significant or had small effect sizes suggests that there is room to select for some of the lower heritability traits as rankings across environments were in general aligned, e.g., in practical plant breeding one can select for lower or higher protein content in wheat despite the well-known observation that the amount of protein in the grain is dominated by the environmental conditions [55]. In general, the expected differences between waxy and non-waxy genotypes were observed, although there were exceptions. For example, the non-waxy genotype DH133783 grouped with the waxy genotypes studied here for high W-SRC and short batter flow (Table 3). Additionally, the waxy genotype DH140490 had a lower-than-expected absolute RVA breakdown grouping it with the non-waxy genotypes until the proportional breakdown was calculated to show DH140490’s proportional viscosity breakdown grouped it with the other two waxy genotypes. A combination of short peak time and high proportional breakdown under the RVA conditions used here appears to be a robust indicator of waxy status.

Although not one of our *a priori* research questions, our observations indicate that breeding for PHS resistance in food barley should be a priority to prevent loss of functionality in locations with higher probabilities of rain near harvest. Our data also highlighted five genotypes, three waxy and two non-waxy, that appeared to be systematically more susceptible to PHS. The suggestion that waxy genotypes may be more susceptible to PHS needs to be examined in future studies using a diverse collection of waxy barleys. PCA suggested that six traits, a manageable number, might effectively characterize basic food barley physicochemical functionality (Figure 1).

## Figures and Tables

**Table 1:** Genotypes, growth habit, seed source, starch type, and color of winter barley samples.

Genotype	Growth Habit	Source	Starch	Color
10.0655	Facultative	OSU ^1^	Waxy	White
10.0662	Facultative	OSU	Waxy	White
DH133529	Facultative	OSU	Non-Waxy	White
DH133535	Facultative	OSU	Non-Waxy	White
AMAZE 10	Winter	Virginia Tech	Non-Waxy	White
VA15H-79 WS	Winter	Virginia Tech	Non-Waxy	White
Buck	Winter	OSU	Non-Waxy	White, Blue
#STRKR	Winter	OSU	Non-Waxy	White, Blue
10.1154	Winter	OSU	Non-Waxy	White
10.1986	Winter	OSU	Non-Waxy	White
10.1492	Winter	OSU	Non-Waxy	White
1_4	Winter	OSU	Non-Waxy	White
DH133783	Winter	OSU	Non-Waxy	White
DH140490	Winter	OSU	Waxy	White
DH140394	Winter	OSU	Non-Waxy	White

^1^ Oregon State University.

**Table 2:** Summary statistics of measured food-barley traits after samples with PHS ^1^ were removed (see also Table S1).

	PHS Samples Removed						
	Min	Max	Range	Mean	SD	N	Missing
Hardness Index	21.6	78.8	57.2	53.2	12.8	161	19
Protein (% d/b)	6.4	18.6	12.2	10.2	2.3	158	22
BG ^2^ (% d/b)	2.9	8.2	5.3	5.1	1.0	159	21
Water SRC	86.2	166	79.9	108	12.3	159	21
Batter Flow (cm)	1.5	16.8	15.3	9.3	3.2	161	19
RVA Peak Viscosity (cP)	3010	6861	3851	4912	726	161	19
RVA Breakdown (cP)	892	4619	3727	2078	566	161	19
RVA Peak Time (min.)	2.9	5.5	2.7	4.7	0.6	161	19
Cooked Grain Hardness (g)	3680	13950	10,270	7139	1901	160	20
Cooked Grain Yield (%)	245	302	57	268	9.6	160	20

^1^ PHS = pre-harvest sprouting; ^2^ BG = mixed linkage beta-glucan.

**Table 3:** Three-way ANOVA for food traits of winter barleys.

	Hardness Index	NIRS Grain Protein (%db)	BG (%db)	Water SRC (%)	Batter Flow (cm)	RVA Peak Viscosity (cP)	RVA Breakdown (cP)	RVA Peak Time (min)	Cooked Grain Yield (%)	Cooked Hardness (g)
3-WAY ANOVA										
*Model r^2^*	0.97 *	0.94 *	0.94 *	0.95 *	0.97 *	0.92 *	0.95 *	0.99 *	0.84 *	0.90 *
*F-values*										
Genotype	44.92 *	8.74 *	74.21 *	58.96 *	89.97 *	29.3 *	63.88 *	503.0 *	17.83 *	13.51 *
Location	700.8 *	13.50 *	0.35ns	199.8 *	503.4 *	16.9 *	79.46 *	31.61 *	0.28ns	63.36 *
Year	37.78 *	156.2 *	25.13 *	5.99 *	95.31 *	29.0 *	87.62 *	40.28 *	12.39 *	79.39 *
Genotype * Location	7.30 *	3.37 *	2.27 ns	5.37 *	4.16 *	6.57 *	5.85 *	6.71 *	2.87 *	1.93 ns
Genotype * Year	4.04 *	3.99 *	3.12 *	4.56 *	6.71 *	8.82 *	7.71 *	3.08 *	2.25 *	1.50 ns
Location * Year	440.5 *	313.6 *	8.27 *	25.04 *	106.3 *	35.3 *	67.97 *	93.48 *	2.75 ns	95.91 *
Genotype *Location *Year	6.32 *	2.57 *	3.10 *	4.78 *	2.89 *	4.89 *	3.60 *	3.58 *	1.25 ns	2.06 ns
Genotype means										
#STRKR	49.2 e	10.2 abc	4.3 de	105.2 bc	10.6 ab	4888 cde	2026 c–f	4.96 abc	268.2 b-e	7879 abc
1_4	46.9 ef	9.5 bc	5.2 bc	106.3 bc	9.6 bc	4884 cde	1855 efg	5.02 ab	277.4 ab	6344 de
10.0655 (Fac, W)	58.4 b	11.4 a	6.8 a	126.4 a	5.0 e	5389 ab	3356 a	3.36 e	264.3 cde	5904 e
10.0662 (Fac, W)	56.7 bcd	10.8 ab	6.8 a	126.6 a	5.0 de	4794 cde	2686 b	3.55 d	252.5 f	7186 a–e
10.1154	51.1 de	10.3 abc	5.2 bc	100.6 bc	10.7 ab	5061 bcd	1761 fg	5.08 a	265.0 cde	6724 cde
10.1492	50.3 e	9.6 bc	4.5 d	102.1 bc	10.8 ab	5182 bcd	2051 cde	5.07 a	259.3 ef	8257 ab
10.1986	48.8 ef	10.2 abc	5.5 b	106.9 b	9.0 c	4552 e	1864 efg	4.98 abc	261.1 ef	7909 abc
AMAZE 10	55.8 bcd	10.3 abc	4.7 cd	99.9 c	10.6 ab	4442 e	1851 efg	4.90 bc	271.8 a–d	6140 de
Buck	43.9 f	9.4 bc	4.0 e	103.7 bc	11.4 a	5197 bc	2201 cd	4.87 c	270.3 a–d	7837 abc
DH133529 (Fac)	60.8 b	11.3 a	5.4 b	105.4 bc	8.9 c	5750 a	2264 c	5.02 ab	264.1 de	6874 b–e
DH133535 (Fac)	51.9 cde	9.9 bc	4.4 de	101.5 bc	11.0 ab	4719 de	1811 efg	4.99 abc	279.4 a	5609 e
DH133783	59.2 b	11.2 a	5.4 b	123.9 a	6.4 d	5374 ab	2207 cd	4.86 c	270.5 a–d	8649 a
DH140394	57.7 bc	10.0 abc	4.2 de	99.7 c	11.5 a	4433 e	1568 g	5.11 a	274.5 abc	7557 a–d
DH140490 (W)	70.9 a	8.8 bc	6.9 a	122.4 a	5.0 e	3688 f	1904 d–g	2.96 f	278.4 ab	5754 e
VA15H-79WS	47.0 ef	10.3 abc	4.4 de	101.2 bc	11.6 a	4517 e	1868 efg	4.96 abc	271.8 a–d	7528 a–d
Location means										
Corvallis	59.6 a	10.5 a	5.1 a	112.5 a	7.9 b	5029 a	1976 b	4.78 a	267.9 a	7677 a
Freeville	46.8 b	10.0 b	5.1 a	102.9 b	10.8 a	4797 b	2179 a	4.69 b	268.8 a	6615 b
Year means										
2018	52.7 b	8.7 b	5.1 b	109.5 a	10.0 a	5095 a	2223 a	4.71 b	266.8 b	6829 b
2019	50.9 c	11.0 a	5.4 a	107.7 ab	8.1 b	4974 a	2171 a	4.68 b	266.4 b	6272 c
2020	55.9 a	11.0 a	4.8 c	106.0 b	9.9 a	4679 b	1850 b	4.81 a	271.6 a	8277 a

Mean values followed by the different letter within each section in a column (genotype, location, or year) are significantly different based on Tukey’s HSD for genotype and year and on Student’s *t* for location. Significance was set at *p* ≤ 0.01. * = significant at *p* ≤ 0.01; ns = not significant *p* > 0.01; Fac = Facultative; W = Waxy.

## Data Availability

Publicly available datasets were analyzed in this study. This data can be found here: https://ir.library.oregonstate.edu/concern/datasets/6w924k89t (accessed on 6 July 2022).

## Supplementary Materials

The following supporting information can be downloaded at: https://www.mdpi.com/article/10.3390/foods11172642/s1, Figure S1: Monthly total rainfall (-) and normal1 rainfall (…) (A,B) and deviation from normal (-) (C,D) for Corvallis (A,C) and Freeville (B,D).; Figure S2: Monthly average temperature (-) and average normal1 temperature (…) (A, B) and deviation from normal (-) (C,D) for Corvallis (A,C) and Freeville (B,D).; Table S1: Description of 6 testing environments over two locations (states) and three growing seasons.; Table S2: Annual rain, normal1 rain, and deviation from normal, and annual average maximum and minimum temperatures and deviation from normal across the three crop seasons covered in the study.; Table S3: Summary statistics of measured traits including samples with pre-harvest sprouting.
