# Supplementary material for: Effect of Genotype and Environment on Food-Related Traits of Organic Winter Naked Barleys
Source: Foods. 2022 Aug 31;11(17):2642. doi: 10.3390/foods11172642 (PMC9455238; doi:10.3390/foods11172642)
Supplement: Supplementary file 1 [file foods-11-02642-s001.zip › foods-1867813-supplementary.pdf]

Table S1 - Description of 6 testing environments over two locations (states) and three growing seasons.

Location	Year	Latitude	Longitude	Planting date	Harvest date	Grain yield $kg.ha^{-1}$	Heading date (DAP)[1]
Corvallis, OR	2018	44.63°N	-123.19°W	2017-10-16	2018-06-30	3037	121
Corvallis, OR	2019	44.56°N	-123.26°W	2018-10-22	2019-07-22	4533	118
Corvallis, OR	2020	44.56°N	-123.26°W	2019-10-11	2020-07-06	4432	115
Freeville, NY	2018	42.50°N	-76.30°W	2017-10-05	2018-07-10	4879	144
Freeville, NY	2019	42.50°N	-76.30°W	2018-10-10	2019-07-11	3839	151
Freeville, NY	2020	42.50°N	-76.30°W	2019-10-10	2020-07-16	3675	146

[1] DAP: days after planting

Table S2 - Annual rain, normal[1] rain, and deviation from normal, and annual average maximum and minimum temperatures and deviation from normal across the three crop seasons covered in the study.

	CORVALLIS			FREEVILLE		
	2018	2019	2020	2018	2019	2020
Crop year total rain (mm)	951	907	759	764	899	828
Crop year normal rain (mm)	1041	1041	1041	779	779	779
Deviation (mm)	-90	-134	-282	-15	+119	+49
Crop year avg temp (°C)	10.5	10.3	10.2	6.0	5.7	6.6
Crop year normal temp (°C)	10.1	10.1	10.1	6.0	6.0	6.0
Deviation (°C)	+0.4	+0.2	+0.1	0	-0.3	+0.6

[1] For Corvallis, normal values are 20-year averages from the weather station at the location. For Freeville, normal values are 30-year averages from Ithaca NY, ~12 km from the plots.
https://agsci.oregonstate.edu/hyslop-weather-station
http://www.nrcc.cornell.edu/wxstation/ithaca/ithaca.html

Table S3 – Summary statistics of measured traits including samples with pre-harvest sprouting.

All samples							
	Min	Max	Range	Mean	SD	N	Missing
Hardness Index	21.6	78.8	57.2	53.2	12.7	179	1
NIRS Protein (% d/b)	6.4	18.6	12.2	10.2	2.2	176	4
BG[1] (% d/b)	2.9	8.2	5.3	5.2	1.1	177	3
Water SRC	86.2	166	79.9	108	12.4	177	3
Batter Flow (cm)	1.5	16.8	15.3	9.2	3.3	179	1
RVA Peak Viscosity (cP)	190	6861	6671	4568	1265	179	1
RVA Breakdown (cP)	156	4619	4463	1963	653	179	1
RVA Peak Time (min.)	2.2	5.5	3.3	4.6	0.8	179	1
Cooked Grain Hardness (g)	3495	13950	10455	6982	1957	178	2
Cooked Grain Yield (%)	245	302	57	269	10	178	2

[1] BG = mixed linkage beta-glucan

Figure S1- Monthly total rainfall (-) and normal[1] rainfall (…) (A, B) and deviation from normal (-) (C, D) for Corvallis (A,C) and Freeville (B,D).

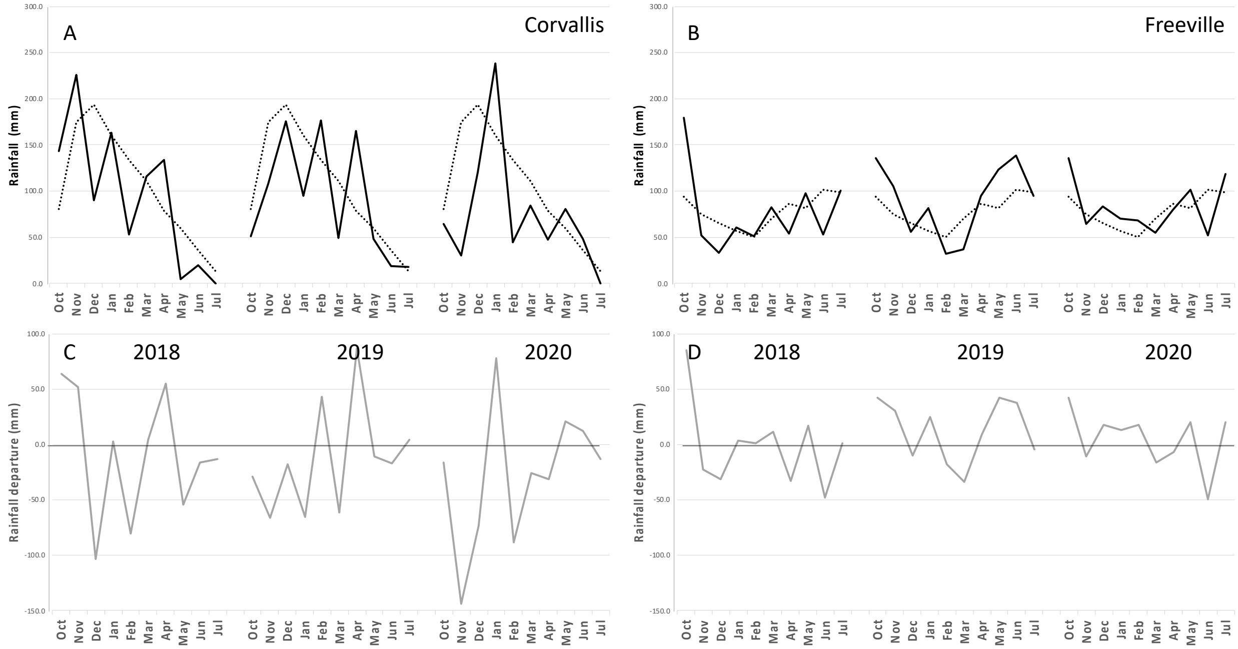

[1] For Corvallis, normal values are 20-year averages from the weather station at the location. For Freeville, normal values are 30-year averages from Ithaca NY, ~12 km from the plots.
https://agsci.oregonstate.edu/hyslop-weather-station
http://www.nrcc.cornell.edu/wxstation/ithaca/ithaca.html

Figure S2- Monthly average temperature (-) and average normal[1] temperature (…) (A, B) and deviation from normal (-) (C, D) for Corvallis (A,C) and Freeville (B,D).

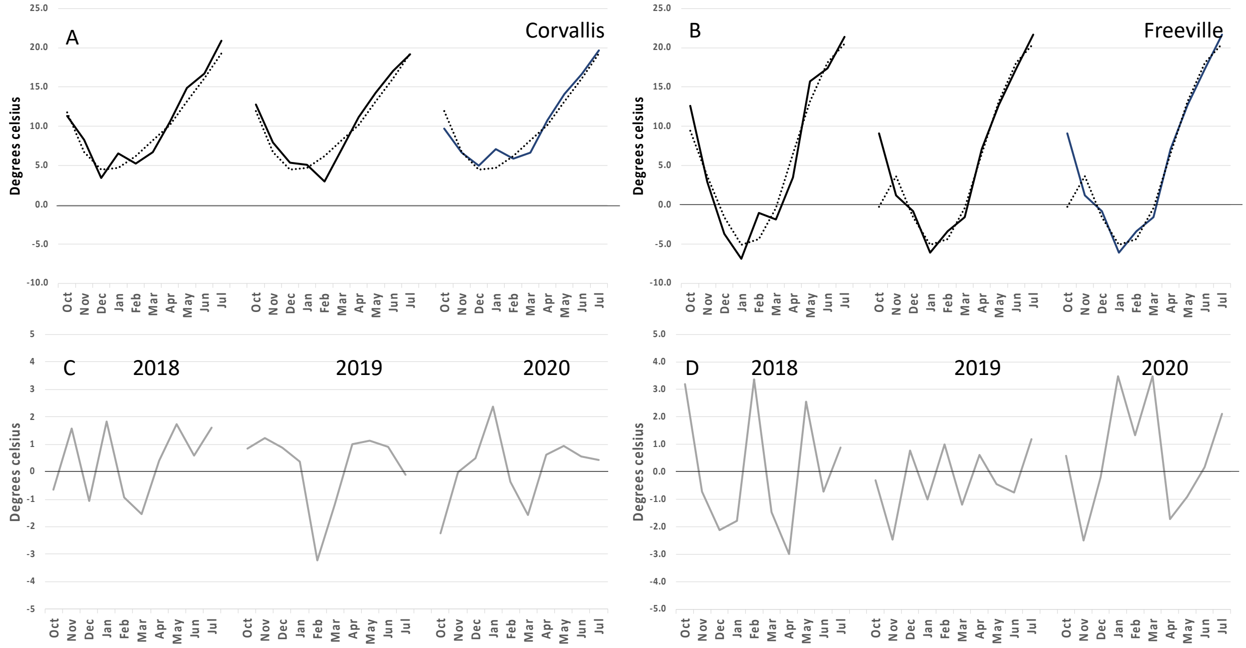

[1] For Corvallis, normal values are 20-year averages from the weather station at the location. For Freeville, normal values are 30-year averages (1990-2020) from Ithaca NY, ~12 km from the plots.
https://agsci.oregonstate.edu/hyslop-weather-station
http://www.nrcc.cornell.edu/wxstation/ithaca/ithaca.html
